# Oleanolic acid attenuates obesity through modulating the lipid metabolism in high‐fat diet‐fed mice

**DOI:** 10.1002/fsn3.4408

**Published:** 2024-08-29

**Authors:** Guangjie Zhang, Huiying Zhang, Ruiyi Dong, Hongmei Zhao, Junfeng Li, Weiming Yue, Zheng Ma

**Affiliations:** ^1^ School of Biology and Food Engineering Anyang Institute of Technology Anyang China; ^2^ Department of Thoracic Surgery Qilu Hospital of Shandong University Jinan China; ^3^ College of Physical Education Hunan Normal University Changsha China; ^4^ College of Food Science and Engineering Jilin University Changchun China

**Keywords:** lipid metabolism, obese mice, oleanolic acid, regulatory effect

## Abstract

As a natural pentacyclic triterpenoid, oleanolic acid has hepatoprotective, anti‐inflammatory, and antioxidant activities. This work performed the in vitro experiments and animal assay to explore whether oleanolic acid alleviates lipid accumulation induced by high‐fat diet by mediating PPARγ. Oil red O staining showed that oleanolic acid can reduce lipid accumulation in HepG2 cells, which were treated with oleic acid and palmitic acid. Immunofluorescence, western blot analysis, and RT‐qPCR showed that oleanolic acid could promote nuclear translocation of PPARγ and reduce the expression level of PPARγ, C/EBP‐β, and SREBP‐1c. The results of in vivo experiments indicated that dietary intervention with oleanolic acid can effectively improve the fat accumulation in liver tissue and attenuate the level of IL‐6 and TNF‐α in serum caused by high‐fat diet. Meanwhile, oleanolic acid did not cause lesions in vital organs at the experimental concentrations. In addition, the computer simulation indicated that oleanolic acid could directly bind to PPARγ with a reasonable and stable docking conformation. The above research results can provide new evidence for oleanolic acid to prevent nonalcoholic fatty liver disease.

## Graphical abstract

In this study, in vitro cellular experiments and in vivo animal assay were performed to explore whether oleanolic acid alleviates lipid accumulation induced by high‐fat diet by mediating PPARγ. The above research results can provide new evidence for oleanolic acid to prevent nonalcoholic fatty liver disease.

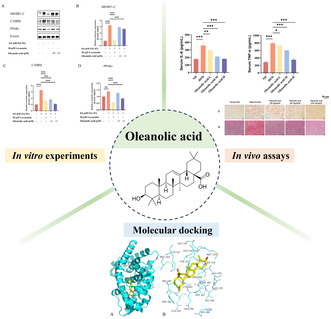

## 1 INTRODUCTION

Excessive intake of high‐fat diet will lead to nutritional imbalance and lipid metabolism disorder. During the past years, metabolic diseases caused by lipid metabolism disorders, including type II diabetes mellitus and obesity, have become a serious global public health problem (Friedman et al., 2018). As a clinical syndrome, nonalcoholic fatty liver disease (NAFLD) is mainly caused by excessive lipid accumulation (Younossi et al., 2018). The pathogenesis of NAFLD is comprehensive and multifactorial, and the lipid metabolism disorder is the main reason for the pathogenesis as well as for the progression of NAFLD (Zhang et al., 2017). Previous studies have shown that abnormal glucose metabolism and lipid metabolism disorders are the basic physiological phenomena caused by high‐fat diet, and high‐fat diet can lead to dysregulation of the expression of genes related to lipid and sugar metabolism (Gao et al., 2020; Li et al., 2019). In addition, the results of animal experiments show that the lipid metabolism of rats and mice is disturbed after feeding a high‐fat diet (Wei et al., 2019; Yaping et al., 2012).To sum up, the reasonable intake with food‐derived bioactive substances has important practical significance for the early prevention of NAFLD caused by lipid metabolism disorders (Mantovani et al., 2021).

As a subset of the nuclear receptor superfamily, the peroxisome proliferator‐activated receptors (PPARs) include three distinct subtypes, namely, PPARα, PPARβ, and PPARγ (Phua et al., 2020). Among them, PPARγ is the main transcription factor regulating adipocyte differentiation, lipogenesis, and glucose metabolism gene expression (Salmeron et al., 2016). PPARγ is associated with adipocyte differentiation; hence, the activation of PPARγ can reduce liver injury as well as fibrosis in mouse models (Alqahtani & Mahmoud, 2016). PPARγ modulates the transcription of the relevant target genes via interacting with the specific peroxisome proliferative response elements. Since these upstream and downstream genes play an important role in the formation of fat, the regulation against them may help to improve lipid metabolism disorders. SREBP1‐C can regulate lipid metabolism by activating PPARγ through endogenous ligand production and controlling the expression of downstream fatty acid synthase (Fang et al., 2019). As a regulator of early adipocytes, the CCAAT/enhancer‐binding protein β (C/EBPβ) plays a role in the induction of PPARγ and SREBP1‐C, as well as in the maintenance of the expression level of SREBP1‐C in the mature adipocytes (Payne et al., 2009). Hence, PPARγ is considered as a promising therapeutic target for addressing metabolic disorders, with its modulatory effect against genes which are involved in lipid metabolism. A considerable number of compounds, such as thiazolidinediones as well as plant extracts, have been used as ligands for PPARγ to regulate lipid metabolism and improve lipid accumulation by targeting PPARγ (Gong et al., 2009; Singh et al., 2023; Zhang et al., 2018).

Some natural phytochemicals extracted from plants show low side effects and therapeutic effects on a variety of diseases. Therefore, they have received extensive attention in the intervention of chronic metabolic diseases. As a natural pentacyclic triterpenoid, oleanolic acid is widely distributed in the olive plants and the quinoa seed coat (Pollier & Goossens, 2012). There is evidence that oleanolic acid may prevent diabetic complications through enhancing insulin response (Yang et al., 2021). In addition, it also has antiviral, antibacterial, antifungal, anticancer, anti‐inflammatory, and antiatherogenic activities (Ayeleso et al., 2017; Castellano et al., 2022). The multiple lines of evidence also suggest that oleanolic acid can also improve the inflammation and oxidative damage caused by diabetes (Iskender et al., 2022).

Previous research has shown that oleanolic acid can regulate lipid metabolism and help improve NAFLD (Luo et al., 2024; Xue et al., 2021). However, it is still unknown whether oleanolic acid can improve lipid disorders by regulating PPARγ. This work performed cellular experiments and animal assay to explore whether oleanolic acid alleviates lipid accumulation induced by high‐fat diet by mediating PPARγ. Finally, the binding mode of oleanolic acid with PPARγ was investigated using molecular docking and molecular dynamics.

## 2 MATERIALS AND METHODS

### 2.1 Chemicals

Fetal bovine serum (FBS) was procured from CLARK Bioscience (Richmond, VA, USA). Oleanolic acid, lovastatin, and palmitic acid were procured from Aladdin (Shanghai, China), while oleic acid was sourced from Macklin (Shanghai, China). 3‐(4,5‐dimethylthiazol‐2‐yl)‐2,5‐diphenyltetrazolium bromide (MTT), rosiglitazone, and GW9662 were acquired from Yuanye (Shanghai, China). Oil red O solution, eosin, hematoxylin, and 4% paraformaldehyde fixation were obtained from Solarbio (Beijing, China). Dulbecco's Modified Eagle Medium (DMEM) was obtained from Gibco (Carlsbad, USA).

The Hifair III First‐Strand cDNA Synthesis SuperMix as well as Hieff qPCR SYBR Green Master Mix were procured from Yeasen (Shanghai, China). The nuclear and cytoplasmic protein extraction kits and bicinchoninic acid (BCA) protein assay kits were supplied by Beyotime (Shanghai, China). TNF‐α ELISA kit and IL‐6 kit were obtained from Cloud‐Clone (Wuhan, China). The serum total cholesterol (TC) and triglyceride (TG) levels were determined by the kits from Jiancheng (Nanjing, China). The anti‐PPARγ antibody and anti‐C/EBP‐β antibody were sourced from Cell Signaling Technology (Danvers, USA). The anti‐SREBP1 antibody was obtained from Abcam (Cambridge, UK). The anti‐GAPDH, anti‐Lamin B1, and anti‐β‐actin antibodies are purchased from Proteintech (Wuhan, China), Santa Cruz (Dallas, USA), and Cohesion Biosciences (Beijing, China). Cohesion Biosciences (Beijing, China) also supplied the goat anti‐rabbit as well as the goat anti‐mouse IgG secondary antibodies.

### 2.2 Cell culture

HepG2 cells were maintained in DDMEM supplemented with 10% fetal FBS and 1% penicillin–streptomycin solution, and in 75 cm^2^ tissue culture flasks. These cells were cultured within a humidified CO_2_ incubator at 37°C with 5% CO_2_, with passaging conducted every 2 days.

### 2.3 Cell viability assay

In this work, the viability of HepG2 cells was assessed by the MTT assay (Ma et al., 2016). HepG2 cells were plated within 96‐well plates with a concentration of 1 × 10^5^ cells/mL. After incubating for 16 h, the growth medium was displaced with a fresh medium dissolving a combination of the oleic acid and palmitic acid (600 μM, 2:1) for 24 h. Subsequently, the medium was displaced with a fresh medium dissolving different concentrations of oleanolic acid. After incubating for an additional 24 h, MTT solution was mixed and then incubated for 4 h, followed by the dissolution of formazan crystals in DMSO.

### 2.4 Oil red O staining and the measurement of the content of TG and TC

HepG2 cells were treated with a combination of oleanolic acid (OA) and palmitic acid (PA) at a concentration of 600 μM with a molar ratio of 2:1 for a duration of 24 h. Subsequently, the cells were subjected to varying concentrations of oleanolic acid (100 and 150 μM) and 50 μM Los for an additional 24 h. Oil red O staining was then performed following the established protocols (Bourebaba et al., 2020). The content of TG and TC was quantified with the commercial kit according to the kit instructions.

### 2.5 PPARγ nuclear translocation assays

HepG2 cells were cultured in 10 cm dishes for 16 h, followed by replacement of the medium with a fresh solution containing RSG, GW9662, and varying concentrations of oleanolic acid for an additional 24 h. The nuclear and cytoplasmic proteins were extracted by the kits, and the levels of PPARγ in the nuclear and cytoplasmic fractions were determined by western blot, with Lamin B1 and GAPDH serving as internal controls for the respective protein fractions.

HepG2 cells were then cultured in confocal Petri dishes and subsequently treated with RSG, GW9662, or varying concentrations of oleanolic acid for immunofluorescence experiments. Following fixation with 4% paraformaldehyde, cell membrane permeabilization was obtained with 0.1% Triton X‐100. The primary antibody targeting PPARγ was then applied and allowed to incubate overnight at 4°C. Subsequently, the HepG2 cells were exposed to the secondary antibody and stained with DAPI for 1 h and 15 min, respectively (Wang et al., 2022). Then, they can be observed under a fluorescence microscope (Olympus NV, Aartselaar, Belgium).

### 2.6 RT‐qPCR assay

Following treatment of HepG2 cells with a mixture of OA and PA (600 μM, 2:1 molar ratio), lovastatin, or varying concentrations of oleanolic acid, the total RNA was extracted from HepG2 cells with the TRIzol reagent and then the RT‐qPCR assay was performed. The primer sequences employed in qPCR are documented in Table S1.

### 2.7 Western blot assay

Following treatment of HepG2 cells with a combination of OA and PA (600 μM, 2:1 molar ratio), lovastatin, or varying concentrations of oleanolic acid, the cells were harvested and subjected to RIPA cell lysate treatment. The resulting supernatant was utilized for western blot assay. Specifically, the protein samples were transferred to the PVDF membranes using a semidry blotter. Subsequently, the membranes were incubated with the primary antibody overnight at 4°C, followed by incubation with the secondary antibody for 1 h. Protein expression quantification was standardized by normalizing it against β‐actin protein expression through the utilization of Image J software.

### 2.8 Animal experimentation

The animal experiments in this work were performed in accordance with the National Institutes of Health Guide for the Care and Use of Laboratory Animals and were authorized by the Animal Care and Ethics Committee of Jilin University. Eight‐week‐old male C57BL/6J mice were procured from Changsheng (Shenyang, China) and allowed a 1‐week acclimatization period. Afterward, these mice were randomly allocated into five groups (*n* = 3), namely, the high‐fat diet (HFD) group, the HFD group supplemented with oleanolic acid at doses of 30 mg/kg/day (oleanolic acid‐L), 60 mg/kg/day (oleanolic acid‐M), and 120 mg/kg/day (oleanolic acid‐H), as well as the normal diet group (ND). After an 8‐week intervention period, these mice were executed. This work collected the blood samples to obtain serum, which were stored at −80°C. In addition, the heart, liver, kidneys, lungs, and spleen were harvested for histological examination using H&E staining.

### 2.9 Measurement of inflammatory factors

The mice were anesthetized using anhydrous ether and then blood was taken from the eyes of the mice. The obtained blood was placed in a centrifuge tube with anticoagulant and centrifuged at 4°C to obtain the supernatant. The content of inflammatory factors TNF‐α and IL‐6 in serum were determined via ELISA utilizing commercially available kits. The test methods were carried out according to the kit instructions, and the absorbance was calculated from the standard curve.

### 2.10 Histopathological examination

After execution of the mice, the mice were dissected and heart, liver, spleen, lung, and kidney tissues were removed, and fixed in 10% formalin solution for 24 h. The tissue specimens were buried in OCT complex and sectioned and subsequently stained with H&E (Ahmad et al., 2017). Furthermore, the liver sections underwent additional staining with ORO solution to facilitate the visualization of hepatic lipid accumulation (Mehlem et al., 2013). The stained samples were subsequently observed using optical microscope.

### 2.11 Molecular docking of PPARγ with oleanolic acid

The binding interaction of PPARγ with oleanolic acid was explored by molecular docking. Firstly, the structure of ligand binding domain of PPARγ (PPARγ‐LBD) with cocrystallized ligand was acquired from Protein Data Bank (ID 3ET3), and then this structure was processed by Chimera 1.11 and AutoDockTools‐1.5.6, respectively (Artis et al., 2009). Afterward, this work developed and optimized the structure of oleanolic acid by Gauss View and Gaussian 09 W, respectively. In order to verify the docking performance herein, the cocrystallized ligand was redocked with PPARγ‐LBD. Then, oleanolic acid was docked with PPARγ‐LBD by AutoDockTools‐1.5.6 and their binding interaction was visualized by PyMOL.

### 2.12 Molecular dynamics simulation for oleanolic acid–PPARγ–LBD complex

The docking conformation of oleanolic acid with the optimal binding free energy with PPARγ‐LBD was chosen to carry out the molecular dynamics simulation. The topology of PPARγ‐LBD was established by the CHARMM36 all‐atom force field. Afterward, the topology of the compound oleanolic acid was established by an CGenFF server. The oleanolic acid–PPARγ–LBD complex was then placed within the cubic water box, then sodium ions were added to gain the charge neutrality. The equilibrations were carried out to get the energy minimization, and then the position restraints on this system were released. Now that this system was at the ideal pressure and temperature, a 50 ns molecular dynamics simulation was carried out by GROMACS 2019. The root mean squared deviation (RMSD) values of the compound oleanolic acid, as well as that of PPARγ‐LBD were both calculated in this work.

### 2.13 Statistical analysis

The data in this work were statistically analyzed with the SPSS 21.0 software and the Student's t‐test was employed for the assessment of statistical significance.

## 3 RESULTS AND DISCUSSION

### 3.1 Effect of oleanolic acid against the lipid‐accumulated HepG2 cells

**FIGURE 1:**
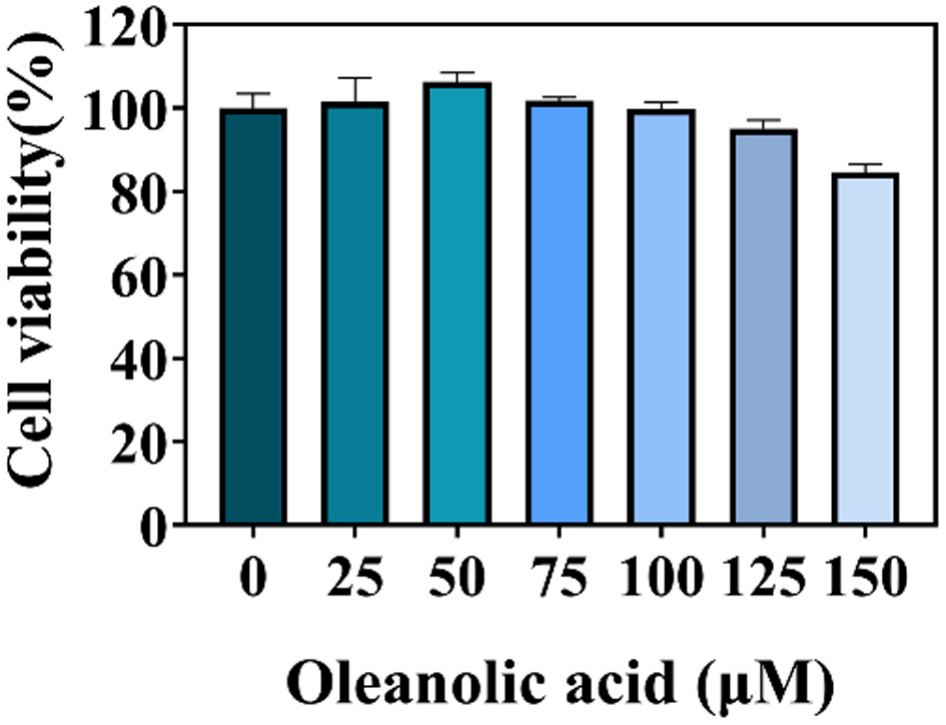
The toxic effects of oleanolic acid on lipid‐accumulated HepG2 cells. The results are expressed as the mean ± standard deviation of three replicates, DMSO control as a control.

The cell survival rate of lipid‐accumulated HepG2 cells treated with oleanolic acid at different concentrations has been shown in Figure 1. Oleanolic acid did not reduce the survival rate of HepG2 cells when the concentration was lower than 100 μM, and even promoted the growth of HepG2 cells when the concentration was 50 μM. When oleanolic acid concentration was 125 and 150 μM, the cell survival rate decreased, but it was still higher than 80%. Based on these results, 100 and 150 μM were chosen as the concentration of oleanolic acid used in the subsequent experiments.

### 3.2 Oleanolic acid inhibited fatty acid‐induced cellular lipid accumulation in HepG2 cells

**FIGURE 2:**
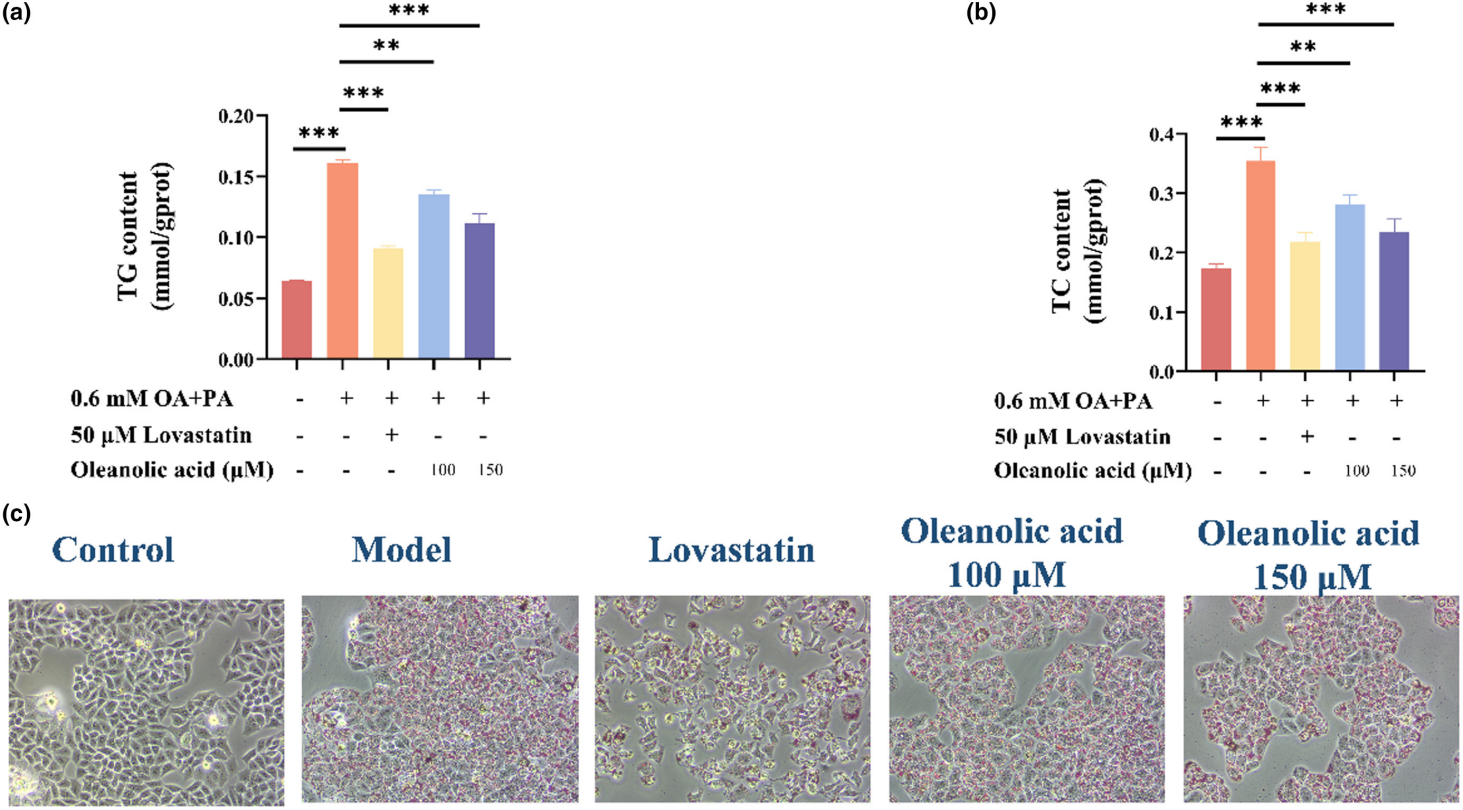
Effects of oleanolic acid on OA‐PA‐induced lipid accumulation in HepG2 cells. (a) TG content measurement in HepG2 cells. (b) Effect of oleanolic acid on TC content in HepG2 cells. (c) Assessment of lipid accumulation in HepG2 cells was performed with oil red O staining. Data are expressed as mean ± SD, *n* = 3. **p < .01, ***p < .001 compared with OA‐PA group.

The oil red O staining technique is presently the predominant method employed for assessing lipid accumulation (Yu et al., 2020). The results of oil red O staining of lipid‐accumulated HepG2 cells treated with oleanolic acid at different concentrations are shown in Figure 2c. It can be seen that the cells in the model group treated with oleic acid and palmitic acid gradually became round and red lipid droplets appeared compared with the control group. After further treatment with lovastatin or oleanolic acid, the red lipid droplets gradually decreased.

The contents of TG and TC in cells of different treatment groups are shown in Figure 2a,b. The cotreatment of oleic acid and palmitic acid significantly increased the contents of triglycerides and total cholesterol in cells (*p* < .001). After lovastatin treatment, the contents of triglycerides and total cholesterol decreased significantly (*p* < .001). Oleanolic acid exhibited a concentration‐dependent reduction in the levels of TG and TC (*p* < .05 at 100 μM and *p* < .01 at 150 μM). Lovastatin is a widely used lipid‐lowering drug (Chu et al., 2019). Established studies reveal that lovastatin could decrease plasma TG level in hypertriglyceridemic patients (Kolovou et al., 2007). In this experiment, lovastatin was regarded as a positive control and oleanolic acid demonstrated comparable effects with lovastatin, which suggested that oleanolic acid can reduce intracellular lipid accumulation.

### 3.3 Oleanolic acid promoted the nuclear translocation of PPARγ

**FIGURE 3:**
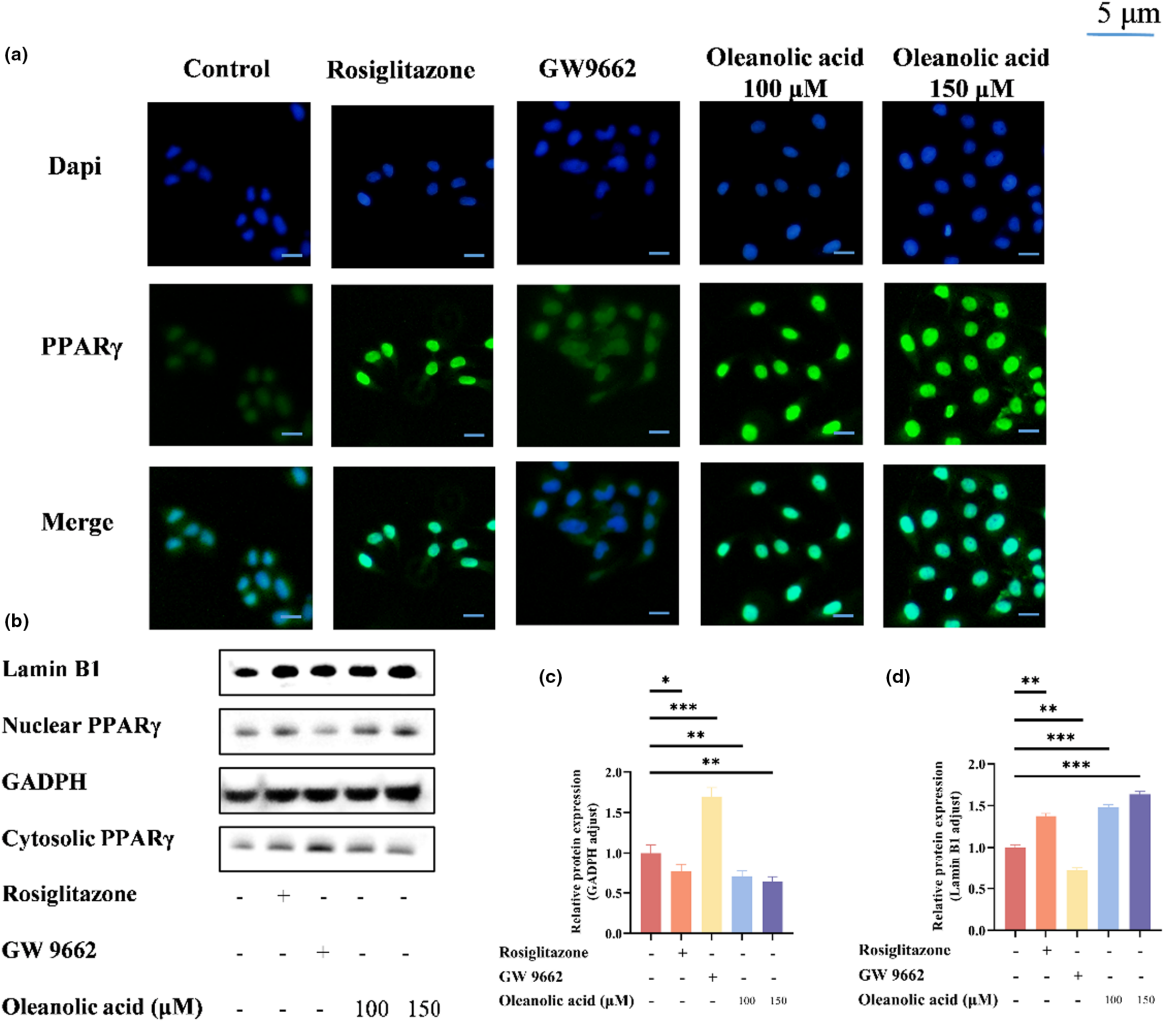
Effects of oleanolic acid on PPARγ nuclear translocation. HepG2 cells were exposed to agonist control (20 μM RSG), antagonist control (20 μM GW9662), or oleanolic acid (100 μM, 150 μM). (a) Nuclear translocation of PPARγ in HepG2 cells was observed by immunofluorescence, green and blue colors represent PPARγ and nucleus, respectively. (b) Western blotting analysis of the nuclear and cytoplasmic PPARγ protein expression levels in HepG2 cells; The scale bar represents 5 μm. (c) Protein expression levels of cytoplasmic PPARγ in HepG2 cells. (d) Protein expression levels of nuclear PPARγ in HepG2 cells. Data are expressed as mean ± SD, *n* = 3. *p < .05, **p < .01, ***p < .001 versus control group.

As a transcription factor regulating adipocyte differentiation and lipogenesis, PPARγ is a crucial factor in the control of lipid metabolism (Iankova et al., 2006). Immunofluorescence and western blot experiments were used to evaluate the effect of oleanolic acid on PPARγ nuclear translocation. The experimental results are shown in Figure 3, in which RSG and GW9662 were agonist and antagonist controls, respectively. The findings from immunofluorescence analysis indicated a significant increase in PPARγ localization within the nucleus of cells treated with rosiglitazone (RSG) and oleanolic acid, as compared to the control group, while PPARγ in the nucleus of GW9662 group decreased. Western blot experiments showed that both RSG treatment and oleanolic acid treatment significantly increased the content of PPARγ in the nucleus and decreased the content of PPARγ in the cytoplasm. This result is consistent with the effect of Ginsenoside Rg1 on PPAR nuclear translocation promotion (Chen et al., 2012), which indicates that oleanolic acid can promote the nuclear translocation of PPARγ.

### 3.4 Oleanolic acid reduced the expression of C/EBP‐β, PPARγ, and SREBP‐1c

**FIGURE 4:**
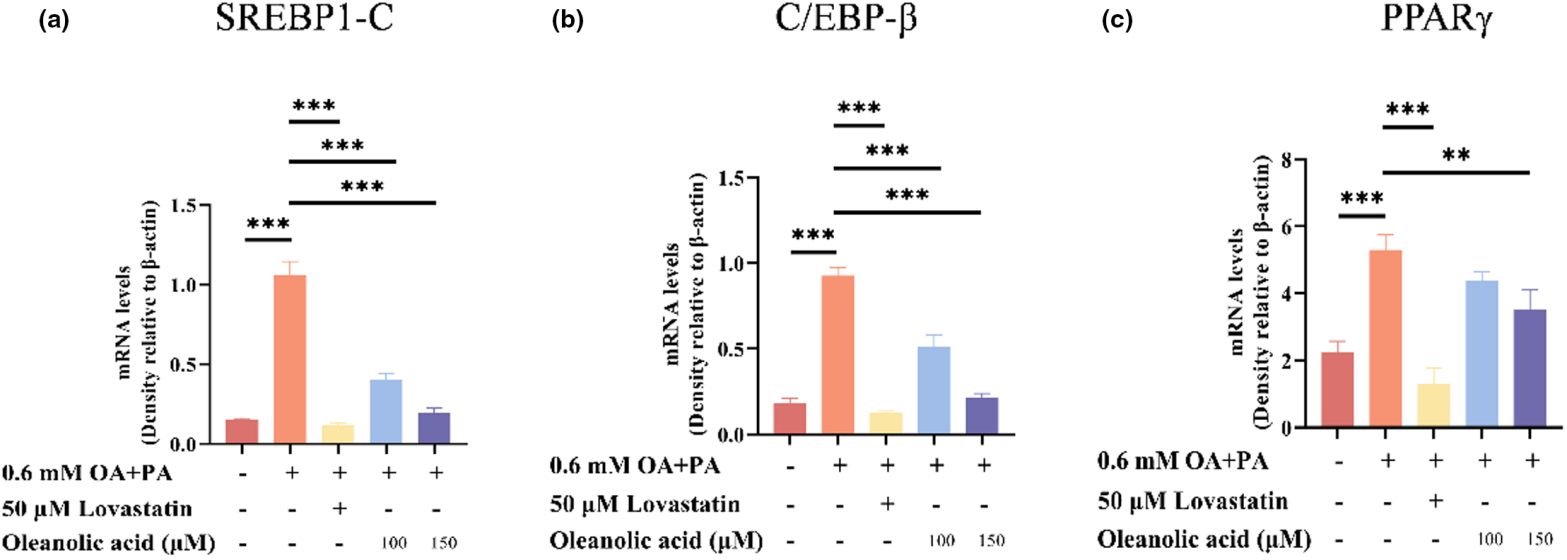
Effects of oleanolic acid on mRNA expression of SREBP1‐C, C/EBP‐β, and PPARγ in lipid‐accumulated HepG2 cells. Data are expressed as mean ± SD, *n* = 3. **p < .01, ***p < .001 versus OA‐PA group.

**FIGURE 5:**
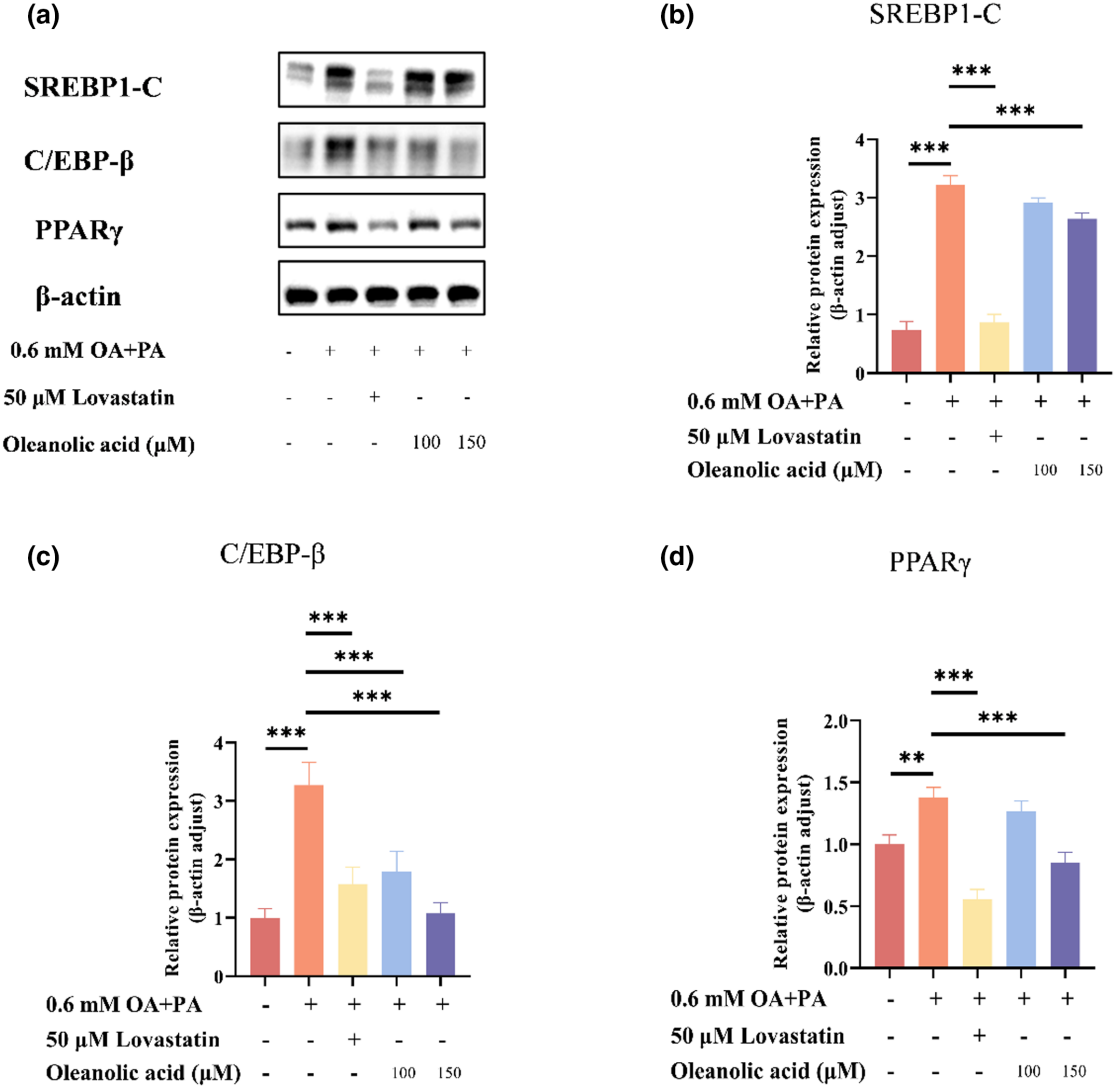
Effects of oleanolic acid on SREBP1‐C, C/EBP‐β, and PPARγ protein expression in HepG2 cells. (a) Protein expression in different experimental groups. (b–d) Protein bands were quantified using image j and normalized to β‐actin expression. Data are expressed as mean ± SD, *n* = 3. **p < .01, ***p < .001 versus OA‐PA group.

C/EBP‐β, PPARγ, and SREBP‐1c are important regulators of lipid metabolism. It is reported that C/EBP‐β can regulate the expression of PPARγ (Lim et al., 2011), and overexpression of PPARγ will lead to lipid accumulation in the liver (Choi, 2019), while SREBP‐1c can promote the synthesis of fatty acids and TG (Guo et al., 2020). The mRNA expression and protein expression levels of C/EBP‐β, PPARγ, and SREBP‐1c in HepG2 cells of different treatment groups are shown in Figures 4 and 5. The results of RT‐qPCR experiments showed that the mRNA expression of C/EBP‐β, PPARγ, and SREBP‐1c increased significantly after the cells were treated with oleic acid and palmitic acid (*p* < .001). At the same time, the results of western blot experiments showed that the protein expression level also increased significantly (*p* < .001). However, after the intervention of lovastatin and oleanolic acid, the mRNA and protein levels of these three regulators decreased significantly. Moreover, the degree of decrease of 150 μM oleanolic acid treatment group was higher than that of 100 μM oleanolic acid treatment group. Previous studies have shown that bisacurone and parthenolide can improve lipid metabolism by reducing the expression of C/EBP‐β, PPARγ, and SREBP‐1c (Annie‐Mathew et al., 2021; Gouthamchandra et al., 2021). The results show that oleanolic acid treatment can reduce the levels of these three regulatory factors induced by oleic acid and palmitic acid, which is consistent with the effect of bisacurone and parthenolide mentioned above. Therefore, it can be inferred that oleanolic acid intervention is beneficial to improve lipid metabolism.

### 3.5 Oleanolic acid reduced the levels of inflammatory factors in the serum of HFD‐fed mice

**FIGURE 6:**
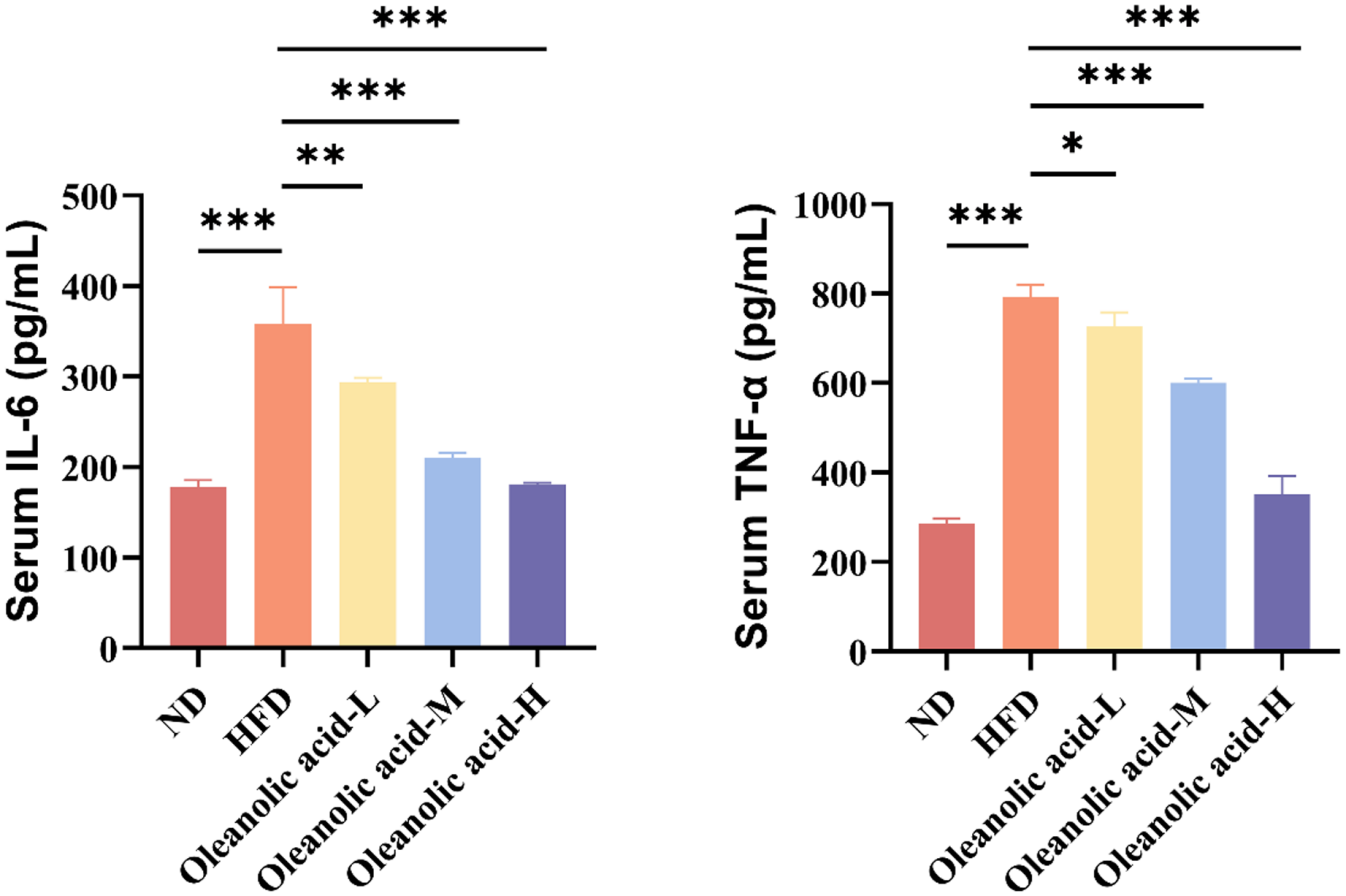
Effects of oleanolic acid on related inflammatory factors in the serum of HFD‐fed mice. (a) Serum levels of interleukin‐6 (IL‐6) in mice. (b) Serum levels of tumor necrosis factor‐α (TNF‐α) in mice. Data are expressed as mean ± SD, *n* = 3. *p < .05, **p < .01, ***p < .001 versus the HFD group.

HFD diet has been shown to elevate serum levels of inflammatory factors in mice, leading to the induction of systemic inflammation (Li et al., 2022) and IL‐6 and TNF‐α are the key inflammatory factors (H. Wang et al., 2015). Figure 6 shows the levels of IL‐6 and TNF‐α in the serum of mice in different treatment groups. It is evident that the serum levels of inflammatory factors in the HFD group were notably elevated compared to those in the ND group, aligning with prior research findings (Wang & Jiang, 2020). Compared with the HFD group, the levels of inflammatory factors were significantly reduced after oleanolic acid intervention (*p* < .01 for IL‐6 and *p* < .05 for TNF‐α), in a dose‐dependent manner. TNF‐α has been reported to increase lipogenesis and lipolysis, leading to abnormal lipid metabolism (Khadke et al., 2019). TNF‐α deficiency could improve hepatic lipid accumulation by increasing adipose tissue storage capacity and reducing hepatic fatty acid uptake and synthesis (Salles et al., 2012). The above results are consistent with those found with Baicalin magnesium. Baicalin magnesium can improve the disorder of lipid metabolism in rats with high‐fat diet and decrease synthesis of TNF‐α and IL‐18 (Guan et al., 2023). The above results showed that oleanolic acid could effectively alleviate the inflammatory response caused by excessive lipid intake in mice.

### 3.6 Oleanolic acid improves liver steatosis in HFD‐fed mice and histopathology examination

**FIGURE 7:**
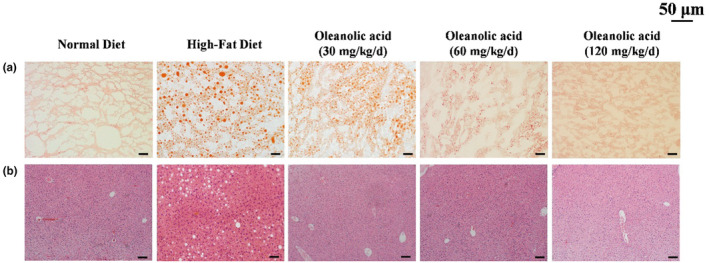
Effects of EA on lipid accumulation in mouse liver tissue. (a) ORO staining of liver fat. (b) H&E staining of liver tissue. Scale lines represent 50 μm.

**FIGURE 8:**
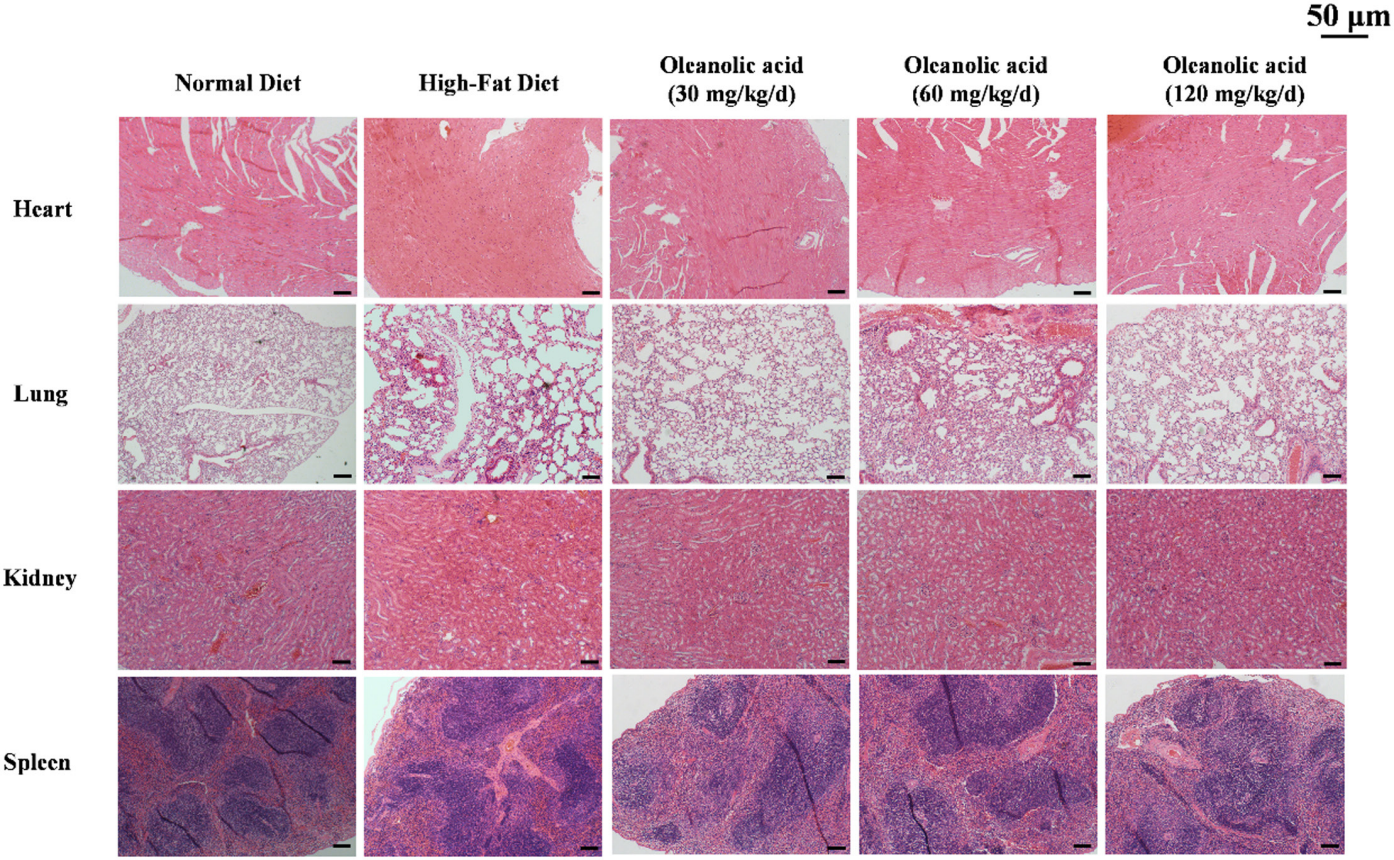
The histopathological sections of heart, lung, kidney, and spleen from each experimental group. Scale bars represent 50 μm.

In order to further investigate the protective effect of oleanolic acid on the liver and its effect on other vital organs, H&E staining and ORO staining were used for pathological analysis of mouse liver tissue, and the results are shown in Figure 7. The H&E staining results of heart, lung, kidney, and spleen are shown in Figure 8. The HFD group showed obvious red lipid droplets as compared with the ND group; however, the size and number of lipid droplets significantly decreased after intervention with oleanolic acid. The results of H&E staining of liver showed that compared with the ND group, the HFD group and oleanolic acid group had different degrees of lipid vacuoles and degeneration. It is worth noting that the lipid vacuoles and denaturation of oleanolic acid group were significantly improved with the increase of oleanolic acid concentration. Meanwhile, as shown in Figure 8, dietary intervention with oleanolic acid did not cause damage or lesions to the heart, spleen, lung, and kidney of mice. The above results show that oleanolic acid can effectively improve the fat accumulation in liver tissue caused by HFD, and at the experimental concentration, it will not cause lesions in vital organs.

### 3.7 The binding interaction of PPARγ with oleanolic acid

**FIGURE 9:**
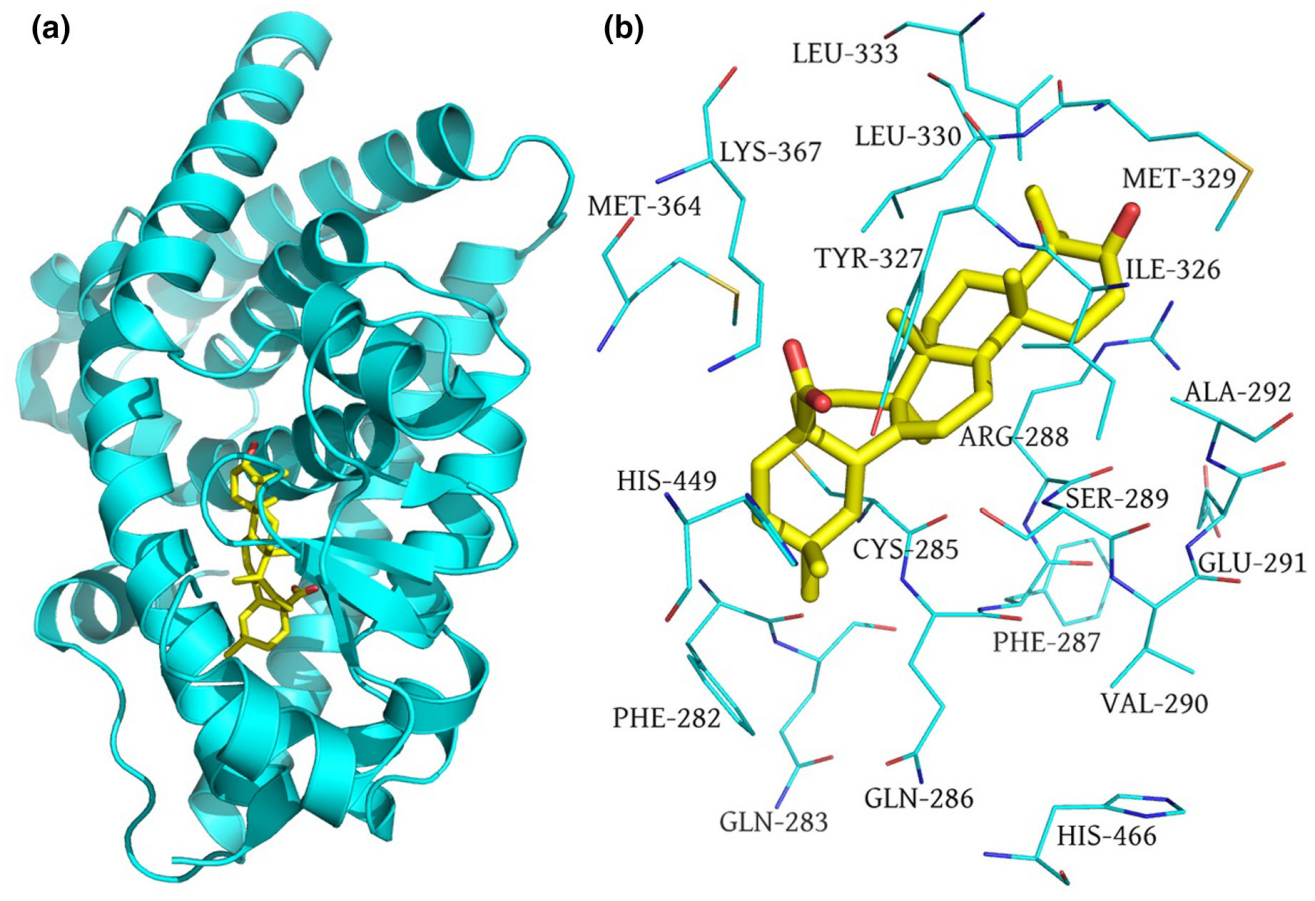
The binding mode of oleanolic acid in the hydrophobic cavity of PPARγ‐LBD (a) and the amino acid residues (cyan lines) located around it within 4 Å (b).

As shown in Figure 9a, oleanolic acid totally entered into the hydrophobic cavity of PPARγ‐LBD and bound within the hydrophobic pocket. Oleanolic acid formed the hydrophobic interactions with amino acid residues Phe282, Phe287, Val290, Ala292, Ile326, Met329, Leu330, Leu333, and Met364 (Figure 9b). Unfortunately, no hydrogen bonds were observed between oleanolic acid and PPARγ‐LBD. Collectively, these findings suggested that the hydrophobic interactions played a pivotal role in the binding of oleanolic acid with PPARγ‐LBD. The binding free energy of oleanolic acid with PPARγ‐LBD was −6.22 kcal/mol, indicating that oleanolic acid was a natural ligand of PPARγ.

### 3.8 The binding stability of oleanolic acid–PPARγ–LBD complex

**FIGURE 10:**
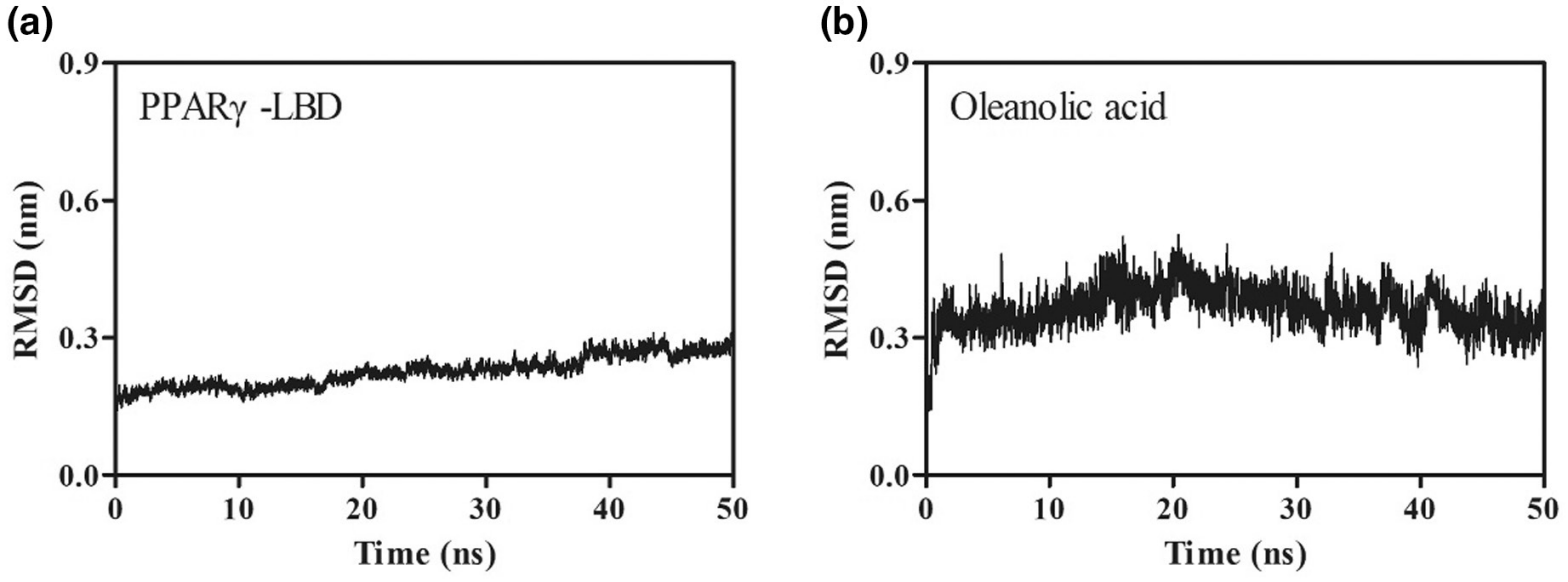
The result of 50 ns molecular dynamics simulation for PPARγ‐LBD (a) and oleanolic acid (b). RMSD, root mean squared deviation.

As shown in Figure 10a, the conformation of PPARγ‐LBD was stable throughout the entire simulation process, with average RMSD values of 0.23 ± 0.03 nm. A similar result was also obtained from the molecular dynamics simulation of oleanolic acid. A minor fluctuation of the conformation of oleanolic acid can be observed in Figure 10b, with the average RMSD values of 0.36 ± 0.05 nm. Hence, the oleanolic acid–PPARγ–LBD complex could maintain stability throughout this 50 ns simulation.

## 4 CONCLUSIONS

This work explored the effect of oleanolic acid on improving lipid accumulation induced by high‐fat diet. Oleanolic acid can reduce lipid accumulation in OA‐ and PA‐treated HepG2 cells via downregulating the gene transcription and protein expression of PPAR‐γ, C/EBP‐β, and Srebp‐1c. Dietary intervention by oleanolic acid can reduce the lipid accumulation in hepatocytes and hepatocyte degeneration, and the level of inflammatory factors in serum of mice fed with high‐fat diet. Furthermore, it has been observed that this intervention does not result in damage to major internal organs at the experimental dosage. The aforementioned research findings can serve as references for further preclinical investigations of the potential of oleanolic acid to mitigate lipid accumulation.

## AUTHOR CONTRIBUTIONS

**Guangjie Zhang:** Investigation (equal); writing – original draft (equal). **Huiying Zhang:** Investigation (equal); writing – original draft (equal). **Ruiyi Dong:** Data curation (equal); investigation (equal). **Hongmei Zhao:** Data curation (equal); investigation (equal). **Junfeng Li:** Data curation (equal); investigation (equal). **Weiming Yue:** Project administration (equal); writing – review and editing (equal). **Zheng Ma:** Project administration (equal); writing – review and editing (equal).

## CONFLICT OF INTEREST STATEMENT

The authors declare that they have no known competing financial interests or personal relationships that could have appeared to influence the work reported in this paper.

## Supporting information


Table S1.

## Data Availability

The data that support the findings of this study are available on request from the corresponding author.
