# Supplementary material for: Oleanolic acid attenuates obesity through modulating the lipid metabolism in high‐fat diet‐fed mice
Source: Food Sci Nutr. 2024 Aug 29;12(10):8243–54. doi: 10.1002/fsn3.4408 (PMC11521747; doi:10.1002/fsn3.4408)
Supplement: Supplementary file 1 — Table S1. [file FSN3-12-8243-s001.docx]

**Table. S1** Amplification primer sequences for HepG2 cell in RT-qPCR.

| Gene | Forward (5ʹ-3ʹ) | Reverse (5ʹ-3ʹ) |
| --- | --- | --- |
| *SREBP1-C* | *CTCCCTAGGAAGGGCCGTA* | *GCCGACTTCACCTTCGATGT* |
| *C/EBPβ* | *GCACAGCGACGAGTACAAGA* | *AGCTGCTCCACCTTCTTCTG* |
| *PPARγ* | *CCAGAAGCCTGCATTTCTGC* | *TGATCCCAAAGTTGGTGGGC* |
| *β-actin* | *CTCACCATGGATGATGATATCGC* | *ATAGGAATCCTTCTGACCCATGC* |
